# Supplementary material for: Characterization of the Role of Extracellular Vesicles Released from Chicken Tracheal Cells in the Antiviral Responses against Avian Influenza Virus
Source: Membranes (Basel). 2021 Dec 31;12(1):53. doi: 10.3390/membranes12010053 (PMC8780788; doi:10.3390/membranes12010053)
Supplement: Supplementary file 1 [file membranes-12-00053-s001.zip › membranes-1515743-supplementary/Figure S1.pdf]

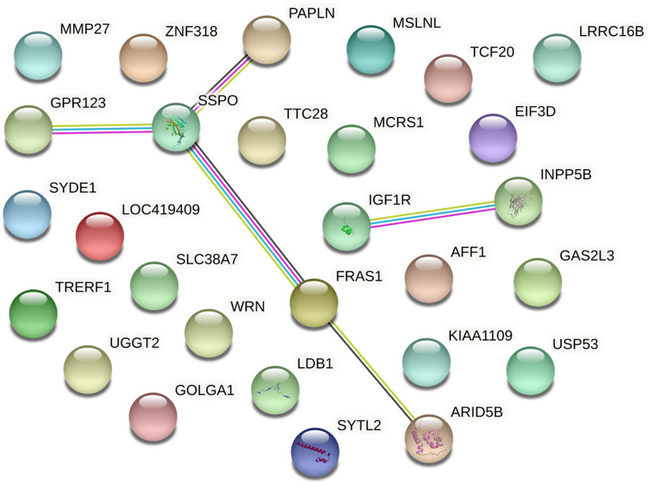
MMP27
ZNF318
PAPLN
MSLNL
TCF20
LRRC16B
GPR123
SSPO
TTC28
MCRS1
EIF3D
SYDE1
LOC419409
IGF1R
INPP5B
SLC38A7
AFF1
GAS2L3
TRERF1
FRAS1
WRN
UGGT2
KIAA1109
USP53
LDB1
GOLGA1
ARID5B
SYTL2

(**a**)

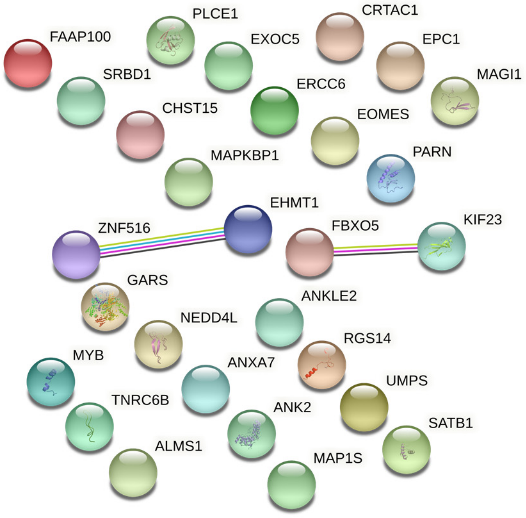
PLCE1
CRTAC1
FAAP100
EXOC5
EPC1
SRBD1
MAGI1
ERCC6
CHST15
EOMES
MAPKBP1
PARN
EHMT1
ZNF516
FBXO5
KIF23
GARS
ANKLE2
NEDD4L
RGS14
MYB
ANXA7
UMPS
TNRC6B
ANK2
SATB1
ALMS1
MAP1S

(**b**)

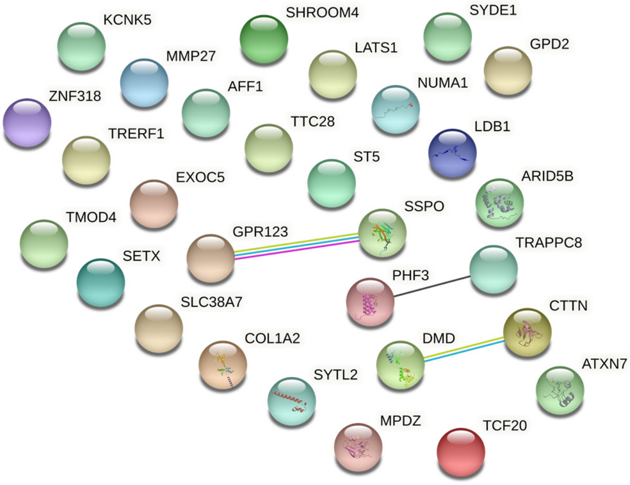
KCNK5
SHROOM4
SYDE1
GPD2
LATS1
MMP27
AFF1
NUMA1
ZNF318
TTC28
LDB1
TRERF1
ST5
EXOC5
ARID5B
SSPO
TMOD4
GPR123
TRAPPC8
SETX
PHF3
SLC38A7
CTTN
COL1A2
DMD
ATXN7
SYTL2
MPDZ
TCF20

(**c**)

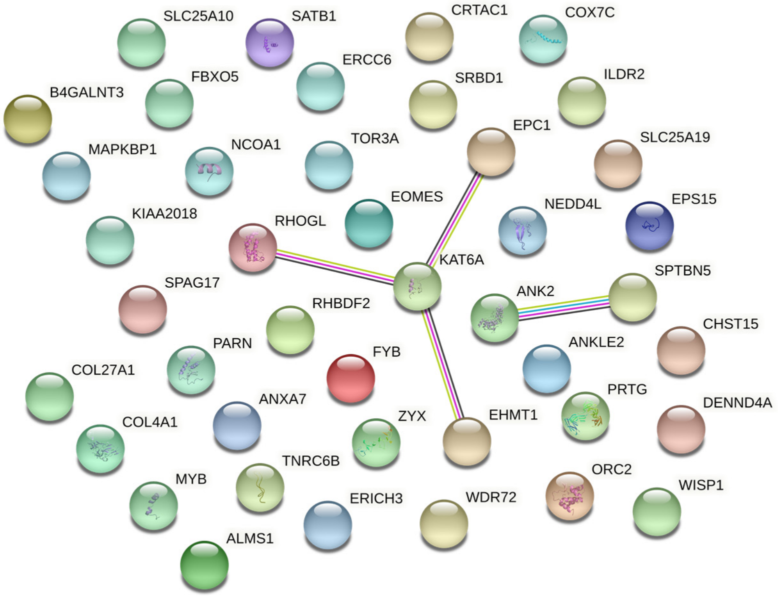
SLC25A10
SATB1
CRTAC1
COX7C
ERCC6
FBXO5
SRBD1
ILDR2
B4GALNT3
EPC1
MAPKBP1
NCOA1
TOR3A
SLC25A19
EOMES
NEDD4L
EPS15
KIAA2018
RHOGL
KAT6A
SPTBN5
ANK2
SPAG17
RHBDF2
CHST15
ANKLE2
PARN
FYB
COL27A1
PRTG
ANXA7
DENND4A
COL4A1
ZYX
EHMT1
TNRC6B
ORC2
MYB
WDR72
WISP1
ERICH3
ALMS1

(d)

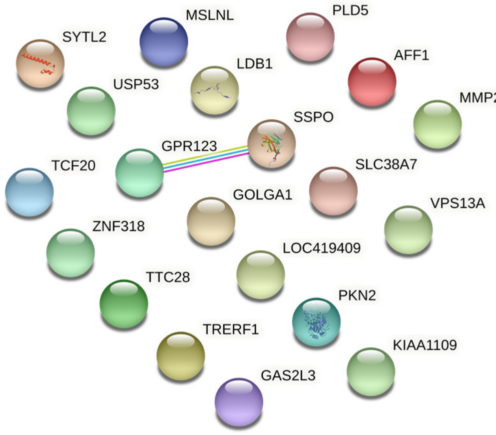
MSLNL
PLD5
SYTL2
LDB1
AFF1
USP53
MMP27
SSPO
GPR123
TCF20
SLC38A7
GOLGA1
VPS13A
ZNF318
LOC419409
TTC28
PKN2
TRERF1
KIAA1109
GAS2L3

(e)

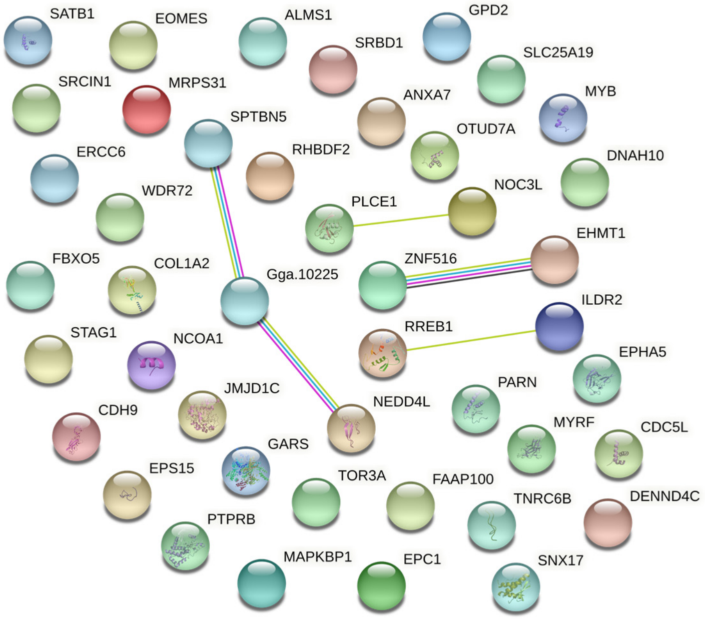
SATB1
EOMES
ALMS1
GPD2
SRBD1
SLC25A19
SRCIN1
MRPS31
ANXA7
MYB
SPTBN5
OTUD7A
RHBDF2
ERCC6
DNAH10
WDR72
NOC3L
PLCE1
EHMT1
FBXO5
COL1A2
ZNF516
Gga.10225
ILDR2
RREB1
STAG1
NCOA1
EPHA5
JMJD1C
PARN
NEDD4L
CDH9
MYRF
CDC5L
GARS
EPS15
TOR3A
FAAP100
TNRC6B
DENND4C
PTPRB
MAPKBP1
EPC1
SNX17

(f)

Figure S1. Network analysis of protein-protein interactions among AIV upregulated. (**a**), AIV down-regulated (**b**), LPS up-regulated (**c**), LPS down-regulated (**d**), polyI:Cup-regulated (**e**), and polyI:C down-regulated (**f**) groups of EVs from TOCs, obtained from STRING database, where blue edges represent known interactions from curated databases, purple edges represent experimentally determined known interactions, yellow edges represent interactions from textmining, and black edges represent interactions from co-expression.
